# Effect of sex hormones, garlic and fennel extracts in layers’ breeders diet on inherited offspring sex

**DOI:** 10.1371/journal.pone.0338813

**Published:** 2026-07-09

**Authors:** Zeinab Bardel, Ali Asghar Saki, Asghar Mirzaie-Asl, Pouya Zamani

**Affiliations:** 1 Department of Animal Science, Faculty of Agriculture, Bu-Ali Sina University, Hamedan, Iran; 2 Department of Plant Biotechnology, Faculty of Agriculture, Bu-Ali Sina University, Hamedan, Iran; Ain Shams University Faculty of Agriculture, EGYPT

## Abstract

This study aimed to examine the effect of dietary supplementation with sex hormones, garlic, and fennel extracts in layer breeders on the molecular sex ratio of their progeny. One hundred layer breeders, aged sixty-five weeks, were assigned to five treatments with five replicates of four hens each in a completely randomized design (CRD) for five weeks. The experimental treatments consisted of: (1) a control diet (corn and soybean meal-based), (2) control diet + testosterone (1 mg/kg), (3) control diet + progesterone (1 mg/kg), (4) control diet + fennel extract (400 mg/kg), and (5) control diet + garlic extract (400 mg/kg). In the third and fifth weeks of the experiment, blood samples were collected from the wing vein of layer breeders to measure sex hormone levels. At the end of the fifth week, eggs were gathered over two consecutive days and incubated at 37.5°C. The results suggest that fennel extract increased serum testosterone levels compared to the control throughout the study period (*P* = 0.057). Garlic and fennel extracts and progesterone increased the female sex ratio, while testosterone treatment increased the male sex ratio compared to the control (*P* = 0.056), although these differences were not statistically significant (*P* > 0.05). The experimental treatments significantly influenced the percentage of embryos produced (*P* < 0.05). No significant effect was observed on blood glucose levels (*P* = 0.076), though numerical differences were noted. Treatment 2 (testosterone) resulted in the lowest female sex ratio and blood glucose levels, whereas treatments 3 (progesterone), 4 (fennel extract), and 5 (garlic extract) yielded the highest female sex ratios and blood glucose levels. These findings suggest that the treatments’ effects may be linked to their impact on blood glucose levels. The results indicate that dietary interventions affect the offspring sex ratio.

## Introduction

Efficiency could be significantly enhanced by controlling offspring sex in the poultry industry. In the layer industry, approximately half of all chickens are culled post-hatching because they are male, a practice deemed wasteful yet economically necessary due to the lack of market for male layer chickens, which neither lay eggs nor exhibit the rapid growth and large breast muscles of broiler breeds [1,2]. Consequently, male chickens are considered a byproduct of egg production and are euthanized immediately after hatching. Manipulating hen reproduction to produce only female offspring could increase productivity, reduce waste, improve profitability, and address animal welfare concerns [2].

In birds, males are the homogametic sex (ZZ) and females are the heterogametic sex (ZW), making the female bird the determinant of offspring sex. The mechanism by which female birds influence offspring sex remains unclear, despite extensive research on the evolutionary and ecological implications [3,4]. An unidentified factor complicates the intricate process of sex determination in birds. Moreover, it remains challenging to identify the variables that influence sex and sex ratio [5–7].

Substantial evidence suggests that female birds can manipulate offspring sex ratios prior to fertilization. Across various bird species, females adjust sex ratios in response to social, environmental, and parental factors [8]. Navara [9] identified food quality, season, stress, female condition, laying order, latitude, and mate quality as key influences on offspring sex ratios. Recent studies suggest that maternal steroid hormones regulate sex ratio determination [2,10]. Sex hormones have been shown to alter sex ratios in birds, and their impact on gender in poultry has been investigated [11].

Research has demonstrated a relationship between offspring sex genetics and plasma hormone levels in hens during sex determination. Pinson [12] reported that maternal steroids can affect sex chromosome segregation. Several studies have identified testosterone as a regulator of sex ratios in birds, though the timing and mechanism of its influence remain unclear [2]. In local Iraqi breeder hens, corticosterone level variations directly influence primary and secondary sex ratios oppositely, impacting offspring sex and inversely affecting hatching rates, as stress reduces the hatching percentage [6]. Corticosterone, progesterone, and testosterone could alter sex ratios at elevated or reduced levels before ovulation [6,12–14]. Supplementation with prednisolone (5, 10, and 20 mg/kg feed) in broiler breeders (Arbor-Acres Plus) shifted initial sex ratios toward males, with the most significant effect at 20 mg/kg [7]. Studies have reported that elevated corticosterone concentrations correlate with increased progesterone levels in blood, contributing to sex hormone imbalances [15]. The timing and quantity of hormones can affect offspring sex ratios [11].

Phytoestrogens are biologically active plant-derived compounds structurally similar to 17-beta-estradiol, capable of binding to estrogen receptors and exerting estrogenic or antiestrogenic effects [16]. Lignans, a class of phytoestrogens, are present in garlic [17]. Coumarin in fennel exhibits aromatase inhibitory, estrogenic, reductase inhibitory, and antiandrogenic activities, while flavonoids and anethole in fennel also display estrogenic properties [18].

Studies have shown that medicinal herbs can influence sex hormones, including estrogen, progesterone, and testosterone. Diets supplemented with fennel have been reported to affect sex hormone levels in animals [19], with fennel extract reducing estrogen and increasing progesterone levels in female rats [20]. Genistein implants in local Iraqi chickens increased blood estrogen and the female offspring sex ratio [21]. In-ovo injection of garlic extract altered sex ratios in broiler chickens and exerted positive effects [22,23] while 200 mg of dry garlic extract in broiler diets increased plasma estrogen levels [23]. However, in-ovo garlic injection had no effect on offspring sex ratios in broilers [24]. Fennel extract treatment influenced serum progesterone in female rats and serum estrogen and testosterone in male rats [25]*.* These effects likely involve interactions among multiple hormones and physiological systems. Stress and elevated corticosterone levels are known to suppress reproduction [26].

Several mechanisms have been proposed to explain how female birds manipulate offspring sex ratios through hormones. These include: 1) Selective follicle atresia [27], occurring in follicles prior to meiotic segregation [28]; 2) Asynchronous follicular development [29], where maternal hormones influence follicle development, though the mechanism for sex-specific differentiation remains unclear [2]; and 3) Non-random segregation of sex chromosomes, considered the most likely mechanism for altering sex ratios [2,9,30]. It has been reported that hormones probably affect chromosome separation through the release of intracellular calcium, which is involved in altering the structure of tubulin, actin, and the cytoskeleton [31,32]. Additionally, steroid hormones (estrogen, progesterone, and androgens) regulate telomerase activity and chromosome length via cell cycle regulators, receptors [33], and gene pathways linked to meiotic segregation [34], thereby influencing sex ratios.

Numerous studies have explored the impact of hormone injection on offspring sex ratios in birds, including [6,13,14,27]. Fewer studies have demonstrated that dietary components can influence sex ratios [7,35]. Modifying diets to alter sex ratios is more practical than hormone injections. Given that the effect of hormones on offspring sex ratios remains unclear and few studies have explored the impact of sex hormones and medicinal plants in diets on bird sex ratios. Therefore, this study investigates the effects of sex hormones and garlic and fennel extracts in the diet of layer breeders on offspring sex ratios.

## Materials and methods

This study was approved by the Research Ethics Committee of Bu-Ali Sina University, Hamedan, Iran, under approval ID IR.BASU.REC.1402.056.

### Experimental animals and diet

This study utilized 100 birds from W80 layer breeders strain, aged 65 weeks, assigned to five treatments with five replicates per treatment, each containing four hens. Two roosters were assigned to each treatment at the start of the experiment. A natural insemination method was used. Roosters were rotated among replicates every two days, with the exchanges occurring simultaneously. The average body weight was similar across replicates, and a 15: 9 hour light: dark cycle was maintained.

Initially, steroid hormone doses (0.25 and 0.75 mg/kg of feed) were evaluated in a preliminary trial to confirm no adverse effects on performance or blood hormone levels in layer breeder hens. Subsequently, a higher dose (1 mg) was selected and tested for its effect on offspring sex ratios.

The experimental treatments consisted of: 1) a control diet (corn and soybean meal-based); 2) control diet + 1 mg/kg testosterone (testosterone undecanoate); 3) control diet + 1 mg/kg progesterone (medroxyprogesterone acetate); 4) control diet + 400 mg/kg fennel extract; 5) control diet + 400 mg/kg garlic extract.

To ensure homogeneity, garlic and fennel extracts were pre-mixed with a small portion of feed before being blended into the entire ration. Testosterone and progesterone were dissolved in oil before incorporation into the feed.

The garlic and fennel extracts used in this study were procured from the Academic Center for Education, Culture, and Research, Karaj, Iran. Testosterone was produced by Organon Company, France, and progesterone was produced by Iran Hormone Company, Tehran, Iran.

The amino acid profiles of corn, soybean meal, and barley were analyzed using Near-Infrared Reflectance Spectroscopy (NIRS). Standardized ileum digestible (SID) amino acid contents were calculated using coefficients provided by Degosa Evonik, Tehran, Iran. Experimental diets were then formulated based on the amino acid profiles, following the W80 layer breeder catalog recommendations (Table 1).

**Table 1:** Ingredients and nutritional composition of experimental diets.

Feed ingredients	Control (kg)	Calculated analysis	Value
Corn	60.82	Metabolizable energy (Kcal/kg)	2750
Soy bean meal	18.34	CP (%)	13.43
barley	7.44	Calcium (%)	4.04
Soy oil	1.00	Available Phosphorus (%)	0.38
Di-calcium phosphate	1.58	Sodium (%)	0.16
Calcium carbonate	9.50	Chlorine (%)	0.16
Salt	0.190	Lysine (%)	0.6300
Sodium bicarbonate	0.230	Methionine (%)	0.3070
Mineral premix^1^	0.350	Methionine + cysteine (%)	0.5100
Vitamin Premix^2^	0.350	Threonine (%)	0.4560
Vitamin D3	0.050	Tryptophan (%)	0.1369
Vitamin E	0.050	Arginine (%)	0.8250
Dl-methionine	0.100	Valine (%)	0.5926

1: Vitamin A 9000000 IU, vitamin D3 2000000 IU, vitamin E 20000 IU, vitamin K3 2500 mg, vitamin B1 2000mg, vitamin B2 6100 mg, Niacin 24500 mg, B6 3000 mg, B9 1000 mg, B12 14 mg, Biotin 100 mg, anti-oxidant 1000 mg, Choline chloride250000mg, carrier up to 2500g, 2: Mn720000mg, Fe 84000 mg, Cu 6000 mg, Zn 53000 mg, I 870 mg, Se 180 mg, Choline chloride250000mg, Carrier up to 2500g,

### Performance

At the end of each week, residual feed in each cage was weighed, fresh feed was supplied to the layer breeders, and feed intake was calculated on a hen-day. Eggs were collected and weighed daily. Performance traits, including feed intake, egg mass, production percentage, and average egg weight, were determined using the following formulas [36]:


$$\begin{array}{c} DFI\;=\;WFI/\;BD \end{array}$$

$$\begin{array}{c} BD\;=\;\text{7}\;\times \;NH\;+\;DR \end{array}$$

$$\begin{array}{c} DFI\;=\;WFI\;/\;BD \end{array}$$

The following abbreviations are used: *BD* (bird-day), *NH* (number of hens), *DR* (number of days the rooster was presented in the pen), *DFI* (daily feed intake), and *WFI* (weekly feed intake of the pen).


$$\begin{array}{c} HD\;=\;\text{7}\;\times \;NH \end{array}$$

$$\begin{array}{c} PP\;=\;NE\;/\;HD\;\times \;\text{1}\text{0}\text{0} \end{array}$$

$$\begin{array}{c} EW\;=\;TWE\;/\;NE \end{array}$$

$$\begin{array}{c} EM\;=\;EW\;\times \;PP \end{array}$$

$$\begin{array}{c} FCR\;=\;DFI\;/\;EM \end{array}$$

The following abbreviations are defined: *HD* denotes hen-day, *NH* represents the number of hens, *PP* indicates production percentage, *NE* refers number of eggs, *EW* signifies average egg weight, *TWE* denotes total weekly egg weight, *EM* represents egg mass, and *FCR* indicates feed conversion ratio.

### Blood parameter

During the third and fifth weeks of the experimental period, the same bird from each cage was selected to monitor changes in hormone levels (estrogen, progesterone, and testosterone). Blood samples (2 mL) were collected from the wing vein, and the serum was separated and stored at −20˚C until analysis. Sex hormone concentrations were quantified using ELISA kits, following the manufacturer’s instructions.

After five weeks, two hens from each replicate were selected, and blood samples were collected from their wing veins into heparinized tubes. The samples were centrifuged at 2500 rpm for 15 minutes to separate the plasma. Blood parameters (glucose, triglyceride, aspartate aminotransferase, alkaline phosphatase, calcium, total protein, magnesium, high-density lipoprotein, low-density lipoprotein, cholesterol) were then analyzed using a chemistry analyzer with Pars Azmun kits (DiaSys Diagnostic Systems GmbH Company).

### Fertility assessment

**Fig 1:**
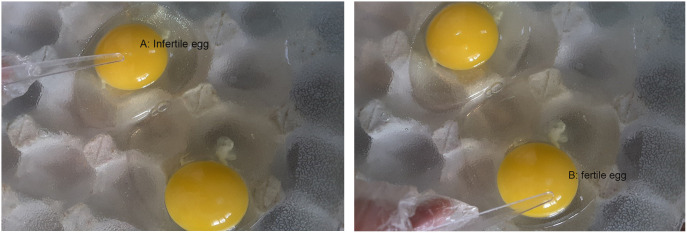
Assessment of egg fertility. **(A)** Unfertilized egg, displaying a white spot on the yolk. **(B)** Fertilized egg, characterized by a white spot on the yolk encircled by a white ring.

Throughout the 5-week experiment, two eggs per replicate were selected weekly. Each egg was broken on a flat surface, and fertilization was assessed as described in Fig 1. Unfertilized eggs exhibited a white spot on the yolk, whereas fertilized eggs displayed a white spot encircled by a white ring.

### Embryonic development

Following the five-week experiment, 110 hatchable eggs were collected over two days and incubated at 37.5 ˚C with 60% relative humidity. On day 18, the eggs were removed from the incubator, and the embryos were carefully extracted, cleaned of residual material, and placed in labeled containers. The embryos were then stored at −20°C until DNA extraction.

### DNA extraction and molecular sexing

**Fig 2:**
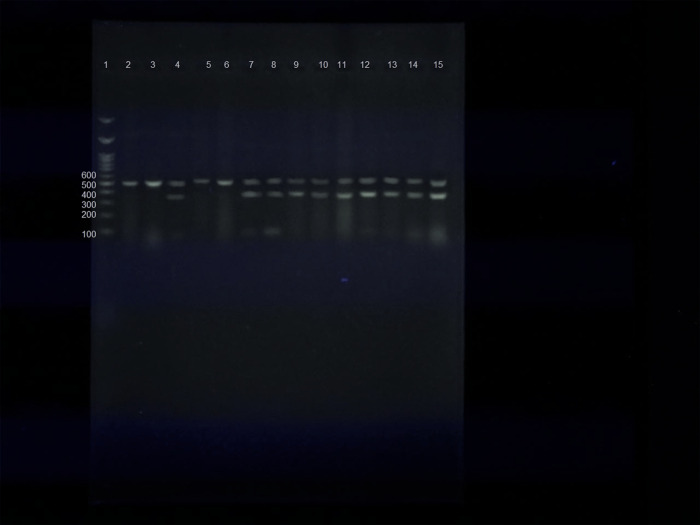
Visualization of DNA bands using a gel documentation system. Lane 1: DNA ladder. Lanes 2, 3, 5, and 6: Male embryo DNA with a 500 bp band. Lanes 4, 7, 8, 9, 10, 11, 12, 13, 14, and 15: Female embryo DNA with 500 bp and 350 bp bands.

Embryos were retrieved from a −20˚C freezer, and 20 mg of tissue (pectoralis muscle) was immediately excised using a scalpel. The tissue was ground in liquid nitrogen using a mortar and pestle. DNA was extracted using the Sina Pure DNA Kit (SinaClon Company, Karaj, Iran), and its concentration was determined via NanoDrop. For polymerase chain reaction (PCR) amplification of the CHD1 gene, 1 μl of nucleic acid (adjusted based on concentration) was used with the specific SF/SR primer pair (SF:5’AGTGCATTGCAGAAGCAATATT-3’, SR:5’ GCCTCCTGTTTATTATAGAATTCAT-3’) [37]. Following a 2-minute denaturation at 94°C, CHD1 gene fragments were amplified using a thermal touchdown protocol: 2 cycles of 94°C for 30 s, 60°C for 30 s, and 68°C for 60 s; 2 cycles of 94°C for 30 s, 57°C for 30 s, and 68°C for 60 s; 2 cycles of 94°C for 30 s,53°C for −30 s, and 68°C for 60 s; and 30 cycles of 94°C for 30 s, 50°C for 30 s, and 68°C for 60 s, followed by a final elongation step at 68°C for 5 minutes [30]. The CHD1 gene fragments, 350 base pairs (CHD1-W) and 500 base pairs (CHD1-Z), were amplified from the W- and Z-chromosomes, respectively, corresponding to female (ZW) and male (ZZ) chicken chromosomes [37]. Subsequently, 6 μl of PCR products were electrophoresed on a 1% agarose gel, and bands were visualized under UV light using a gel documentation system (Fig 2).

### Statistical analysis

This experiment was conducted in a completely randomized design (CRD) with five treatments, five replicates, and four hens per replicate. Data on sex determination, incubation, and fertilization were compared using a Two-way chi-square test, while other data collected during the study were analyzed using the GLM procedure in SAS software.

Performance data, including feed intake, feed conversion ratio, egg mass, production percentage, and egg weight, along with plasma hormone levels (estrogen, progesterone, and testosterone) collected throughout the experiment, were analyzed as repeated measures in a completely randomized design (CRD) using the following statistical model:


$$\begin{array}{c} Y_{ijk}=\;\mu +T_{i}+W_{j}+T_{i}\times W_{j}+{Ea}_{ik}+{Eb}_{ijk} \end{array}$$

Where *Y*_*ijk*_ represents the dependent variable for treatment i*,* week j, and replicate k; *µ* is the overall mean; *T*_*i*_ is the effect of treatment i; *W*_*j*_ is the effect of week j; *T*_*i*_ × *W*_*j*_ is the interaction effect between treatment i and week j; *Ea*_*ik*_ is the mean error; *Eb*_*ijk*_ is the residual effect, with i, j and k = 1, 2, 3, 4, 5.

Blood parameters, including glucose, triglyceride, AST, alkaline phosphatase, calcium, total protein, magnesium, HDL, LDL, and cholesterol, were analyzed in a completely randomized design using the following model:


$$\begin{array}{c} Y_{ij}=\;\mu +T_{i}+e_{ij} \end{array}$$

Where *Y*_*ij*_ is the dependent variable for treatment i, replicate j; *µ* is the overall mean; *T*_*i*_ is the effect of treatment i*;* and *e*_*ij*_ is the mean error.

The normality of residual distributions for production traits and blood parameters was assessed using Kolmogorov-Smirnov and Shapiro-Wilk tests. As residuals for all traits followed a normal distribution, no data transformation was required. Treatment means were compared using Duncan’s multiple range test at a significance level of *P* < 0.05.

## Results

### Offspring sex

The experimental treatments approached statistical significance in altering sex ratio (*P* = 0.056).Testosterone treatment increased the proportion of male offspring, whereas progesterone, fennel extract, and garlic extract increased the female sex ratio in treatments 3, 4, and 5, respectively (Table 2).

**Table 2:** Effects of testosterone (1 mg/kg diet), progesterone (1 mg/kg diet), and garlic and fennel extracts (400 mg/kg diet) on sex ratio in layer breeders.

Experimental treatments	Male percentage	Female percentage
Control	53.33	46.67
Testosterone	69.57	30.43
Progesterone	31.25	68.75
Fennel extract	27.27	72.73
Garlic extract	35.29	64.71
The *p*-value for examining the relationship between the effects of treatments on sex	0.0565	

### Performance

The treatments had no significant effect on performance traits throughout the experimental period (*P* > 0.05). In contrast, egg mass, egg weight, and feed conversion ratio were significantly affected by week (*P* < 0.05) (Table 3).

**Table 3:** Effects of testosterone (1 mg/kg diet), progesterone (1 mg/kg diet), and garlic and fennel extracts (400 mg/kg diet) on layer breeder performance.

Excremental treatment	Feed intake (g/bird/day)	Feed conversion ratio	Egg mass (g/bird/day)	Production percentage (%)	Egg weight (g)
Control	111.56	2.94	39.84	73.05	57.84
Testosterone	111.91	2.91	41.41	74.14	58.73
Progesterone	112.32	2.92	40.12	71.00	60.08
Fennel extract	113.65	2.62	44.17	79.04	59.67
Garlic extract	111.13	2.88	39.36	69.71	59.52
SEM	1.572	0.2144	3.1941	5.7316	1.2292
*P* value					
Treatment	0.8228	0.8628	0.8282	0.8097	0.7183
Week	0.6361	0.0029	0.0009	0.4076	<0.0001
Treatment x Week	0.4290	0.1315	0.0968	0.0271	0.7005

### Fertility and incubation

The experimental treatments had no significant effect on egg fertility (*P* > 0.05). However, treatment 2 (testosterone) exhibited the highest fertilization percentage compared to the control, whereas treatments 3 (progesterone) and 4 (fennel extract) showed the lowest fertilization percentages (Table 4).

**Table 4:** Effects of testosterone (1 mg/kg diet), progesterone (1 mg/kg diet), and garlic and fennel extracts (400 mg/kg diet) on egg fertility and embryo percentage in layer breeders.

	Egg fertility during the rearing		Incubation	
Excremental treatment	Fertile eggs%	Infertile eggs%	Egg without embryo%	Egg with embryo%
Control	90.48	9.52	31.82	68.18
Testosterone	100.00	0.00	0.00	100.00
Progesterone	87.50	12.50	27.27	72.73
Fennel extract	87.50	12.50	50.00	50.00
Garlic extract	88.89	11.11	26.09	73.91
*P* value	0.2689		0.0052	

Experimental treatments significantly influenced embryo percentage (*P* < 0.05). Treatments 2 (testosterone), 5 (garlic extract), and 3 (progesterone) yielded the highest embryo percentages compared to the control, in descending order. Conversely, treatment 4 (fennel extract) exhibited the lowest embryo percentage compared to the control (Table 4).

### Blood parameters

Experimental treatments significantly affected estrogen levels during the study period (*P* < 0.05), with reduced estrogen observed in the second period compared to the first week. Conversely, treatments 2 (testosterone) and 5 (garlic extract) resulted in the lowest testosterone levels, while treatment 4 (fennel extract) yielded the highest testosterone levels (P = 0.057), though no significant difference was found compared to the control (*P* > 0.05) (Table 5).

**Table 5:** Effects of testosterone (1 mg/kg diet), progesterone (1 mg/kg diet), and garlic and fennel extracts (400 mg/kg diet) on plasma hormones in layer breeders.

Experimental treatments	Estrogen (pg/ml)	Progesterone (ng/ml)	Testosterone (ng/dl)
Control	2248.22	3.113	24.350
Testosterone	3153.44	3.029	19.887
Progesterone	3348.80	2.630	22.091
Fennel extract	2902.60	1.919	32.205
Garlic extract	2783.70	3.046	19.593
SEM	515.761	1.153	3.442
*P* value			
Treatment	0.2526	0.9356	0.0570
Week	>0.0001	0.9933	0.0873
Treatment × week	0.2211	0.5750	0.8848

Means denoted by the same letters indicate no significant difference.

Table 6 presents the effects of experimental treatments on blood parameters. Progesterone, fennel, and garlic extracts increased glucose levels, whereas testosterone decreased them compared to the control; however, the highest glucose level, observed with garlic extract, lacked statistical significance (P = 0.076). Calcium levels were significantly elevated by garlic extract and significantly reduced by testosterone, progesterone, and fennel extract compared to the control (*P* < 0.05). Total protein levels increased with garlic extract and testosterone but decreased with progesterone and fennel extract (*P* < 0.05). Magnesium levels rose with garlic extract and testosterone and declined with progesterone and fennel extract, but these changes showed no statistical significance (*P* = 0.08).

**Table 6:** Effects of testosterone (1 mg/kg diet), progesterone (1 mg/kg diet), and garlic and fennel extracts (400 mg/kg diet) on blood parameters in layer breeders.

treatment	Glucose (mg/dl)	Triglyceride (mg/dl)	AST^1^ (U/L)	Alkaline phosphatase (mg/dl)	Calcium (mg/dl)	Total protein (mg/dl)	Magnesium (mg/dl)	HDL^2^ (mg/dl)	LDL^3^ (mg/dl)	Cholesterol (mg/dl)
Control	389.80	1054.60	135.80	361.30	23.76^b^	5.55^ab^	2.98	73.38	16.88	94.94
Testosterone	305.50	734.60	132.80	443.30	17.71^c^	4.22^b^	2.63	69.88	12.89	117.47
Progesterone	468.60	985.70	166.81	438.40	18.23^c^	6.15^a^	3.13	77.38	17.38	127.76
Fennel extract	422.25	1039.38	187.13	387.38	17.48^c^	4.74^ab^	2.51	73.14	14.57	109.95
Garlic extract	555.88	1143.88	177.00	406.50	29.82^a^	6.63^a^	3.31	65.85	11.38	97.00
SEM	54.6780	122.915	32.009	67.168	1.5860	0.3890	0.2130	6.4155	2.8890	19.1860
P value	0.0760	0.1753	0.6893	0.8335	>0.0001	0.0009	0.0808	0.8890	0.5740	0.7095

Means denoted by the same letters indicate no significant difference.

1: Aspartate Aminotransferase (AST); 2: High-Density Lipoprotein (HDL); 3: Low-Density Lipoprotein (LDL)

## Discussion

Although numerous studies have examined laying hen nutrition [20,38,39], its impact on the genetic sex ratio of chickens remains largely underexplored. Investigating the effects of steroid hormones and medicinal plants could offer novel insights into this relationship. Steroid hormones critically regulate genetic and physiological traits in birds [40], while medicinal plants, with their nutritional and therapeutic properties, may influence egg-laying quality [41,42] and, consequently, chicken sex ratios. Thus, analyzing these factors could enhance our understanding of how nutrition and hormones affect the genetic sex ratio of chickens.

Numerous studies, as reviewed by Navara [9], have demonstrated the effects of steroid hormones on the sex ratio of birds. Corticosterone, progesterone, and testosterone at both high and low levels, can influence sex ratios when administered 4–5 hours before ovulation [2,6,13,14,43]. Notably, testosterone injection (1.5 mg per bird) 5 hours before ovulation has been shown to increase the male sex ratio [2]. In Comb White Leghorn laying hens, hormones may affect the sex chromosome contributed by the heterogametic female to the oocyte. However, as testosterone treatments in earlier studies were often administered chronically throughout follicular development or well before the chromosomal segregation, determining their precise impact on offspring sex remains challenging [12]. Wrobel et al. [11] identified corticosterone and testosterone receptors in hen ovarian follicles. Furthermore, evidence suggests that testosterone may enhance meiotic segregation, particularly in the germinal disk region.

The experimental treatments significantly influenced the sex ratio of offspring. Testosterone supplementation increased male offspring to 69.57%, while progesterone, fennel, and garlic extracts elevated female offspring to 68.75%, 72.73%, and 64.71%, respectively. Pinson [12] reported that testosterone injections increased male offspring by 70%, suggesting that testosterone administration during sex chromosome segregation enhances male offspring production. In this study, dietary testosterone similarly elevated the male sex ratio relative to females. Conversely, injecting 2 mg of progesterone several hours before ovulation significantly reduced the male offspring proportion by 25%, compared to 0.25 mg progesterone (61% male) or control oil (63% male) [13]. Jamshidi et al. [24] found that 2–4% tomato powder in broiler breeder diets had no effect on offspring sex. In our study, dietary garlic extract increased the female sex ratio in layer breeders, with progesterone and fennel supplementation similarly elevating female offspring proportions.

### Performance

In this study, performance characteristics, including feed intake, egg mass, production percentage, and average egg weight remained unaffected by the experimental treatments throughout the study period. Mohammadi and Ansari-Pirsaraei [44] reported that testosterone enhances performance and egg characteristics in laying hens toward the end of the laying period. Additionally, growth hormone and testosterone may influence follicular diameters and egg production performance, though these findings contrast with the current study’s results across the entire period. In their study, testosterone was administered via injection at a dose (0.5 mg/kg body weight) different from that used here and was combined with growth hormone.

Progesterone injections were associated with ovarian atrophy and reduced egg production in hens [39]. However, this study observed no such adverse effects from progesterone. This discrepancy may be attributed to the dosage used, which was 2.5 mg daily for 21 days of age in the prior study. Elevated blood progesterone levels have been reported to halt egg-laying in turkeys, even at low doses [45], yet no such effect was observed in this study. The divergence from previous findings may be attributed to the higher progesterone doses (exceeding 0.17, 0.33, 0.5, and 1.5 mg/kg of body weight) administered for 21 days in earlier research, where ovulation ceased within two days of 0.33 mg daily injections. In this study, daily feed intake ranged from 111.13 to 113.65 grams, with daily hormone treatments of 0.111 to 0.114 mg/day.

Phytoestrogen supplementation improved performance and egg quality in quails [46]. Fennel extract, containing estrogen-like compounds, may promote egg production [20]. Research has demonstrated that a diet supplemented with 50 mg/kg of fennel extract improved performance, immune response, and hatchability in post-molted broiler breeder hens, though further studies are required to clarify the underlying mechanisms. Conversely, fennel essential oil at doses of 200, 400, and 600 mg showed no significant effects on laying hen performance [47], nor did a diet with 1% fennel oil [48]. In this study, experimental treatments had no impact on performance. These discrepancies may stem from variations in bird type, fennel extraction method, bird age, and extract dosage.

A meta-analysis demonstrated that garlic significantly affects laying hen performance [42]. Subgroup analysis revealed that garlic extract significantly influenced egg weight, while garlic powder and oil produced similar effects on egg weight. Factors such as country, breed, hen age, garlic preparation form, inclusion levels, number of hens, and treatment duration were identified as predictors of study outcomes. However, a basal diet containing 2.0 g/kg fermented garlic powder showed no significant effect on egg production or egg weight in layer breeder hens. Consequently, the authors suggested that fermented garlic powder at this level could serve as a feed additive for layer breeder diets [38].

### Incubation

The highest embryo percentages were observed in treatment 2 (testosterone), 5 (garlic extract), and 3 (progesterone), respectively, compared to the control group. In contrast, treatment 4 (fennel extract) exhibited the lowest embryo percentage.

Kazemifard et al. [20] reported a linear decrease in hatchability of fertile eggs (from 90% to 86%, *P* = 0.0052) with increasing dietary fennel extract levels. In this study, dietary fennel extract reduced the embryo percentage (from 68.18% to 50%) and egg fertility (from 90.48% to 87.50%), though this reduction showed no statistical significance.

Antol in fennel may exert an estrogenic effect, potentially increasing estrogen levels in males and reducing testosterone levels, which could decrease sperm production in the seminiferous tubules [49]. Although the direct effects of medicinal plants and sex hormones on the percentage of non-embryonated eggs remain underexplored, several factors may contribute. These include advanced laying hen age, the influence of medicinal plants on sex hormones and the reproductive systems [49], and the modulation of plant compounds or hormones in physiological processes [50,51] related to fertilization and embryo formation. Furthermore, the potential impact of medicinal plants on rooster sperm quality warrants further investigation [52]. In this study, the observed decrease in embryo percentage may be attributed to fennel’s effects on the male reproductive system. While no significant difference in egg fertility was observed, this may reflect fennel extract’s adverse impact on egg viability.

Lim et al. [38] reported that dietary garlic supplementation in layer breeder diets increased egg production and hatchability (from 85% to 88%), consistent with this study’s findings (from 68.18% to 73.91%). Garlic supplementation also enhanced sperm vitality and survival in roosters and improved hen egg fertilization rates [38,53]. Musavi et al. [54] demonstrated that garlic boosts sperm production in roosters, regulates testosterone metabolism, and protects sperm from oxidative damage [52]. They hypothesized that garlic improves semen quality parameters, including sperm vitality, and increases male breeder survival rates. These findings indicate that garlic’s positive effects on the male reproductive system may increase embryo production compared to the control group.

Dietary progesterone supplementation increased embryo percentages compared to the control group. Adams [55] highlighted that hatchability decreased with increasing estrogen levels. Estrogen may influence hatchability by affecting calcium metabolism during egg development in the oviduct [50] or through its impact on fat metabolism [20,51]. Both calcium and estrogen are essential for luteinizing hormone (LH) and progesterone production and secretion [56].

### Blood parameters

Recent studies have reported that calcium and magnesium may influence the sex ratio [57]. Therefore, the present study investigated the impact of blood calcium and magnesium levels on sex determination. Our findings revealed that garlic supplementation significantly increased calcium levels, whereas testosterone, progesterone, and fennel extract significantly decreased calcium levels compared to the control group. Although magnesium levels were numerically affected by the experimental treatments, these changes did not reach statistical significance. It has been reported that fennel root powder increases calcium and phosphorus levels in broilers [58] however, in this study using fennel extract in laying hens, calcium levels decreased, and phosphorus remained unchanged. This discrepancy may be attributed to several factors, including the form of fennel used (extract vs. root powder), the dosage administered, and the species or physiological status of the birds (broilers vs. laying hens). Each of these variables can influence the bioavailability and metabolic impact of phytogenic compounds, thereby affecting mineral absorption and regulation differently across experimental conditions.

It has been reported that estrogen may influence calcium metabolism during ovum formation and hormone secretion [51]. In addition to estrogen, our findings suggest that testosterone and progesterone also play a role in modulating calcium levels, as both hormones were associated with a significant decrease in blood calcium concentrations in laying hens. These hormonal effects may reflect complex regulatory mechanisms involved in reproductive physiology and mineral homeostasis. In this study, no significant association was found between blood calcium and magnesium levels and sex determination in layer breeders hens Testosterone injections administered 5 hours before ovulation increased blood testosterone levels, increasing the male sex ratio [12]. Throughout the study, treatments significantly influenced testosterone levels, with the lowest levels in treatments 2 (testosterone), 5 (garlic extract), and 3 (progesterone), and the highest in treatment 4 (fennel extract); however, these differences showed no statistical significance compared to the control group. Estrogen and progesterone levels remained unaffected by the treatments. Previous research indicates that blood testosterone levels in broiler breeders peak 1 hour post-testosterone injection, returning to baseline within 4 hours [59]. Progesterone injections depressed plasma estradiol concentrations in both ad libitum and restricted-fed hens, with plasma testosterone levels significantly lower (*P*  < 0.001) in injected hens compared to non-injected ones [39]. Yang et al. [60] highlighted that aromatase inhibitors affect broiler chicken gender, with estrogen and testosterone levels serving as indicators of sexual differentiation.

Dietary fennel supplementation has been shown to reduce cholesterol levels in birds [48]. Testosterone injections in aged laying hens may increase plasma levels of growth hormone, LDL, HDL, cholesterol, and estradiol [61]. Additionally, garlic supplementation effectively lowers plasma triglycerides, cholesterol, and glucose levels in animals and humans, and reduces egg cholesterol in laying hens [62–67]. Similarly, dietary garlic is associated with reduced blood levels of triglycerides, LDL, and total cholesterol in birds [53,68–70]. Variations across studies may arise from differences in the form of the substance used (e.g., whole seeds, powders, or extracts), the administered dosage, and the species or physiological status of the birds.

The highest plasma total protein levels were observed in laying hens fed diets with 1% garlic powder and 1.5% thyme powder [71]. In this study, total protein levels increased with garlic and testosterone supplementation but decreased with progesterone and fennel extract. No significant effects were observed on HDL, LDL, triglycerides, or cholesterol levels due to the treatments. While some studies report that garlic influences egg production rates, others find no such response, potentially due to variations in preparation methods, product storage, bioactive component concentrations, administered proportions, and study duration [72].

Male sex determination may be influenced by elevated glucose levels in mammals [9,73,74]. It has been reported that an increase in glucose in the maternal bloodstream can impair the blastocysts development in female embryos. This is attributed to the higher mobility of Y sperm, which benefits from increased glucose in cervical mucus during ovulation, facilitating Y-sperm survival and uptake [75].

In mice, circulating glucose levels were reported to influence the sex ratio. when glucose concentrations were experimentally reduced using dexamethasone, fewer male offspring were produced [74]. It has been reported that testosterone significantly affects glucose metabolism, exerting a protective effect in males, but it can have adverse effects in females [76]. Less is known about the relationship between blood glucose levels and sex ratios in birds [9]. In this study, treatments affected plasma glucose levels. Testosterone yielded the lowest glucose levels and the highest male sex ratio, suggesting that reduced glucose concentrations may increase male offspring proportions in birds. Conversely, garlic, fennel extracts, and progesterone resulted in the highest glucose levels compared to the control group, correlating with increased female offspring.

## Conclusion

In the layer poultry industry, culling half of male chickens highlights the need for optimized production. Producing only female chickens could significantly enhance industry productivity. This study investigates dietary manipulation as amethod to alter sex ratios, demonstrating greater practicality and efficiency compared to injection-based approaches. Contrary to expectations of elevated hormone levels, the results revealed a significantly different outcome than predicted.

This study found that testosterone supplementation increased the male sex ratio, whereas progesterone, fennel, and garlic extracts elevated the female sex ratio. These changes were not attributed to alterations in sex hormone levels. Blood glucose levels decreased in the testosterone group but increased in the fennel, garlic, and progesterone groups compared to the control, with the increase approaching statistical significance. The treatments’ effects on sex ratio may be linked to their effect on blood glucose levels. Thus, dietary interventions may modulate sex ratios through unknown mechanisms, possibly related to medicinal plants. Given that the treatments significantly influenced embryo production percentages, further research is needed to clarify the underlying mechanisms.

This study suggests that dietary interventions may enable manipulation of offspring sex ratios. However, further research across diverse avian species is required to assess the feasibility and wider implications of this approach.

## Supporting information

S1 File Raw images: Raw gel data of different treatments were shown in this file. (PDF)

S2 File Raw data: The raw data of the other parameter (sex ration, performance, Fertility and incubation, plasma hormone and blood parameters) were shown in this file. (RAR)
