# Supplementary material for: Effect of sex hormones, garlic and fennel extracts in layers’ breeders diet on inherited offspring sex
Source: PLoS One. 2026 Jul 9;21(7):e0338813. doi: 10.1371/journal.pone.0338813 (PMC13349119; doi:10.1371/journal.pone.0338813)
Supplement: S1 File — (PDF) [file pone.0338813.s001.pdf]

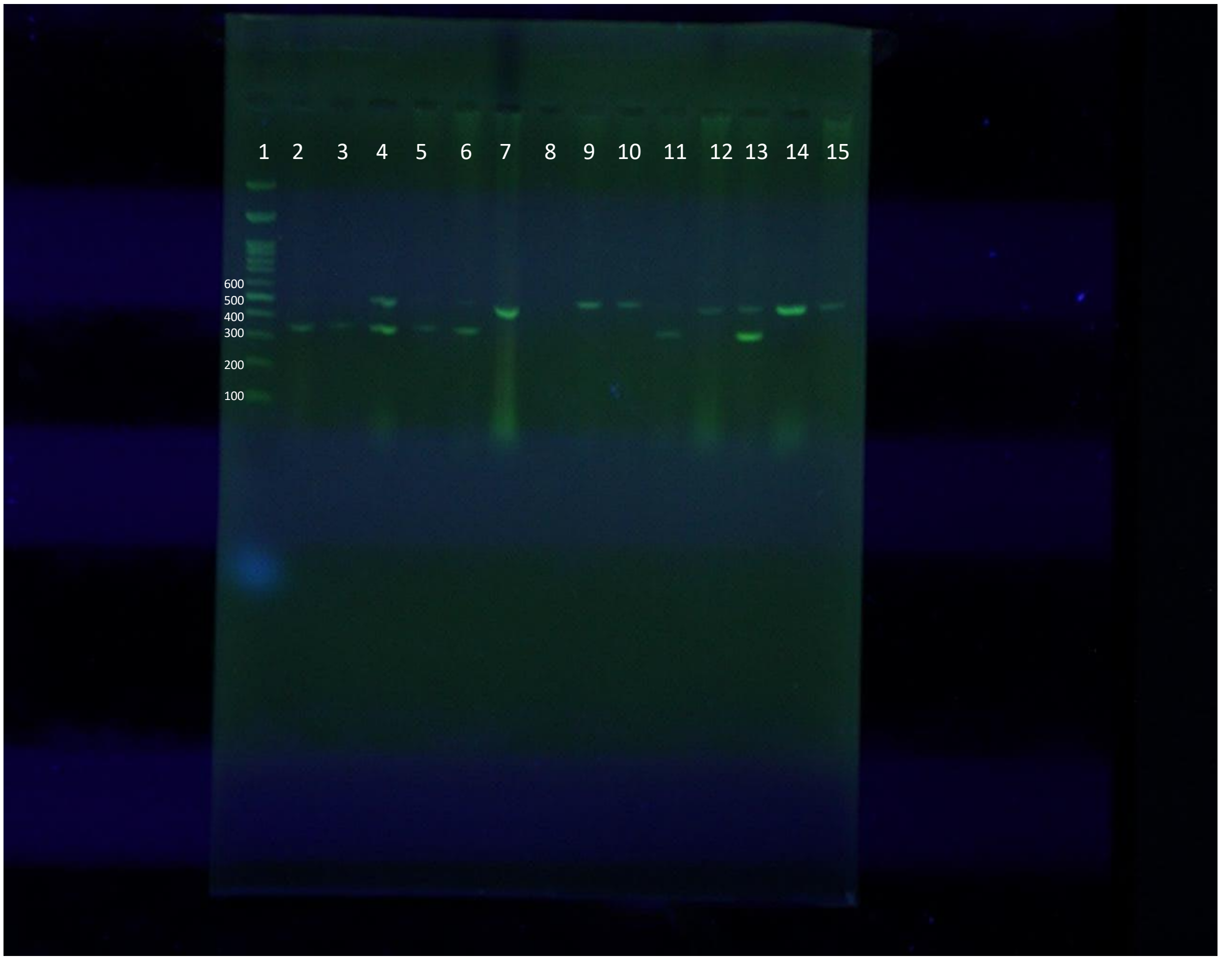


Treatment 1 (Control): DNA Ladder (1). Male embryo: molecular weight of 500 bp (7,9,10,12,14,15), and female embryo: molecular weight of 500 and 350 bp (2,3,4,5,6,11,13).

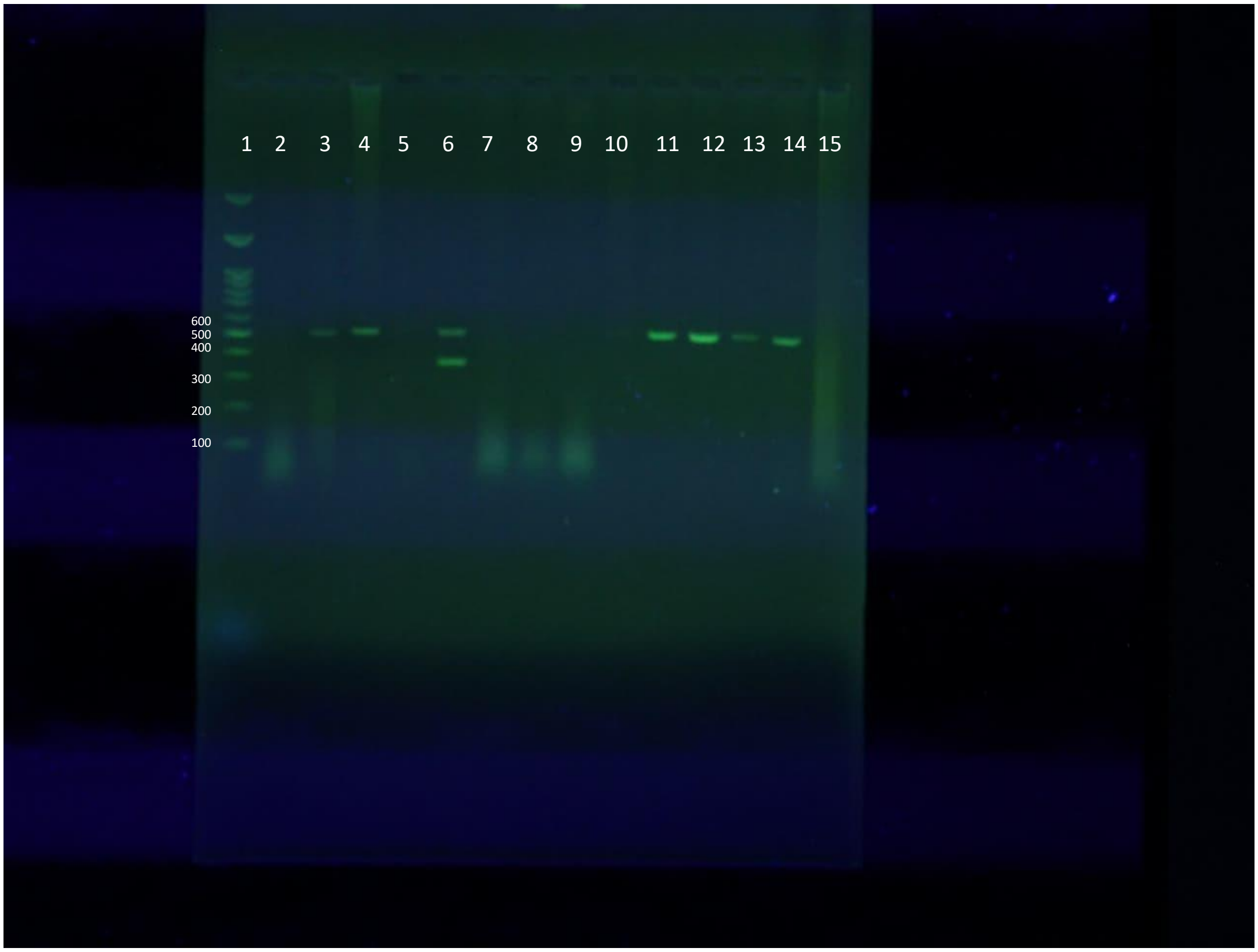


Treatment 2 (Testosterone)- DNA Ladder (1). Male embryo: molecular weight of 500 bp (3,4,5,10,11,12,13,14), and female embryo: molecular weight of 500 and 350 bp (6).

Kjjl

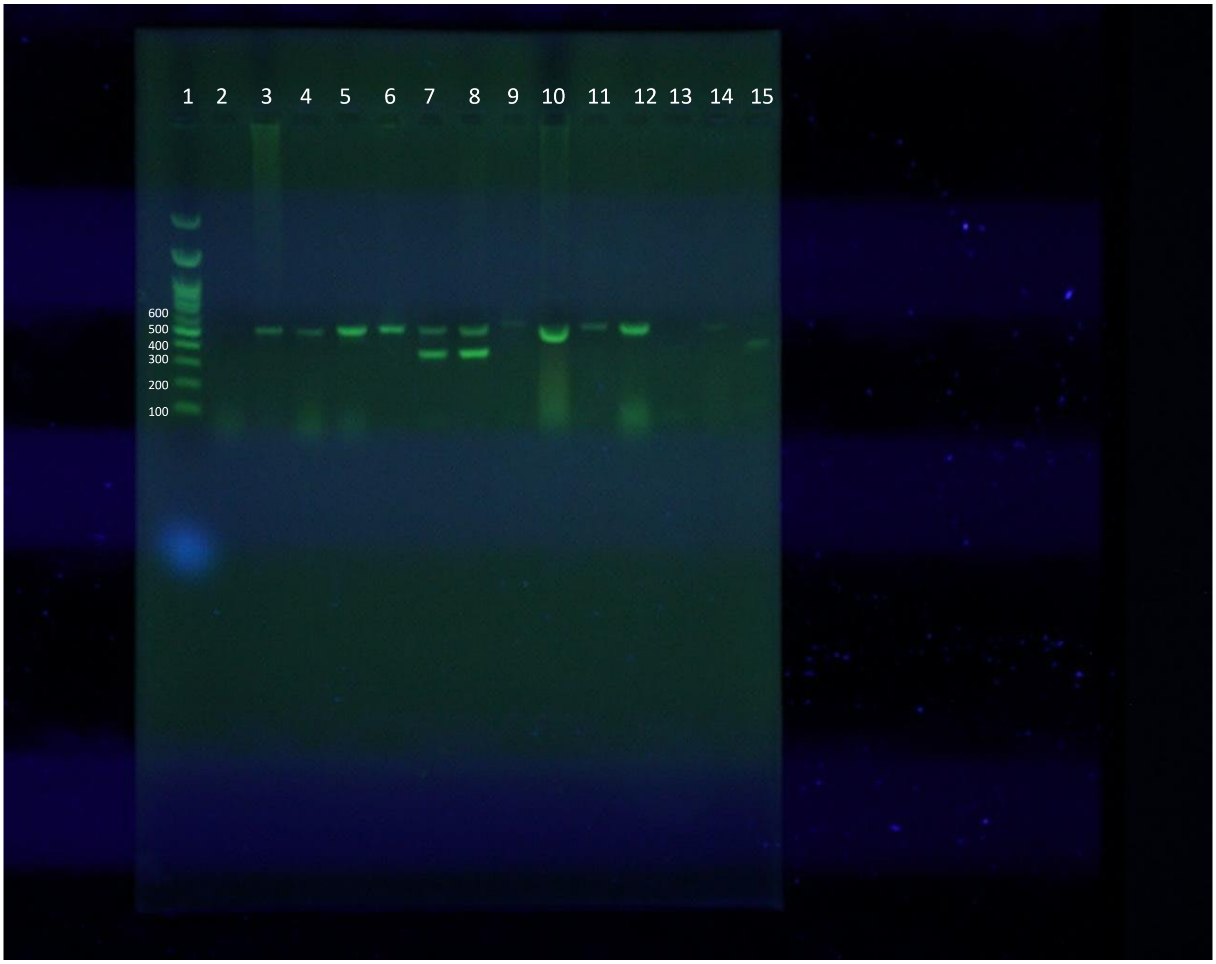


Treatment 2 (Testosterone)- DNA Ladder (1). Male embryo: molecular weight of 500 bp (2,3,4,5,6,9,10,11,12,14), and female embryo: molecular weight of 500 and 350 bp (7,8,13,15).

The samples 9 and 11 are treatment 1(control)

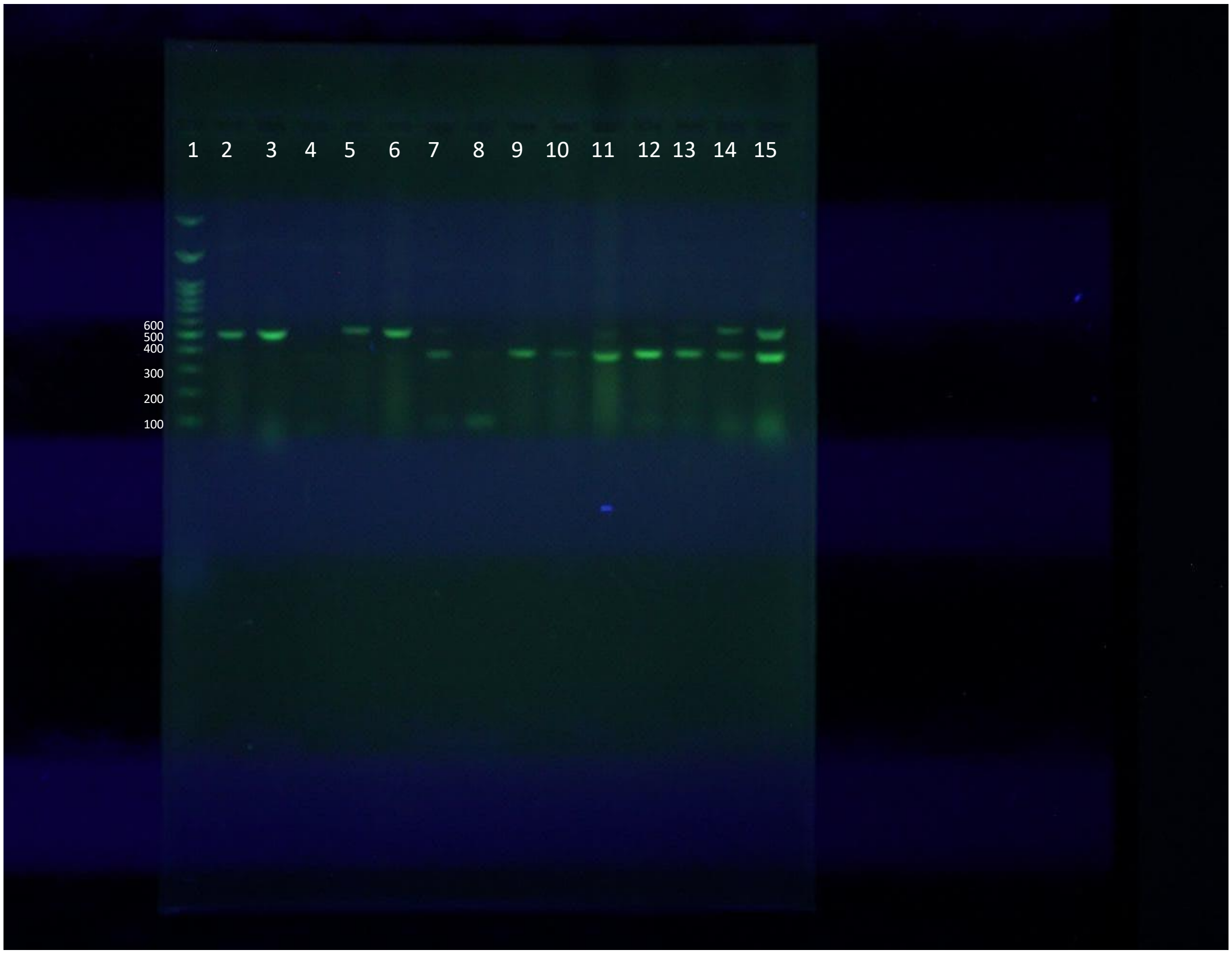


Treatment 3 (progestrone)- DNA Ladder (1). Male embryo: molecular weight of 500 bp (2,3,5,6), and female embryo: molecular weight of 500 and 350 bp (4,7,8,9,10,11,12,13,14,15).

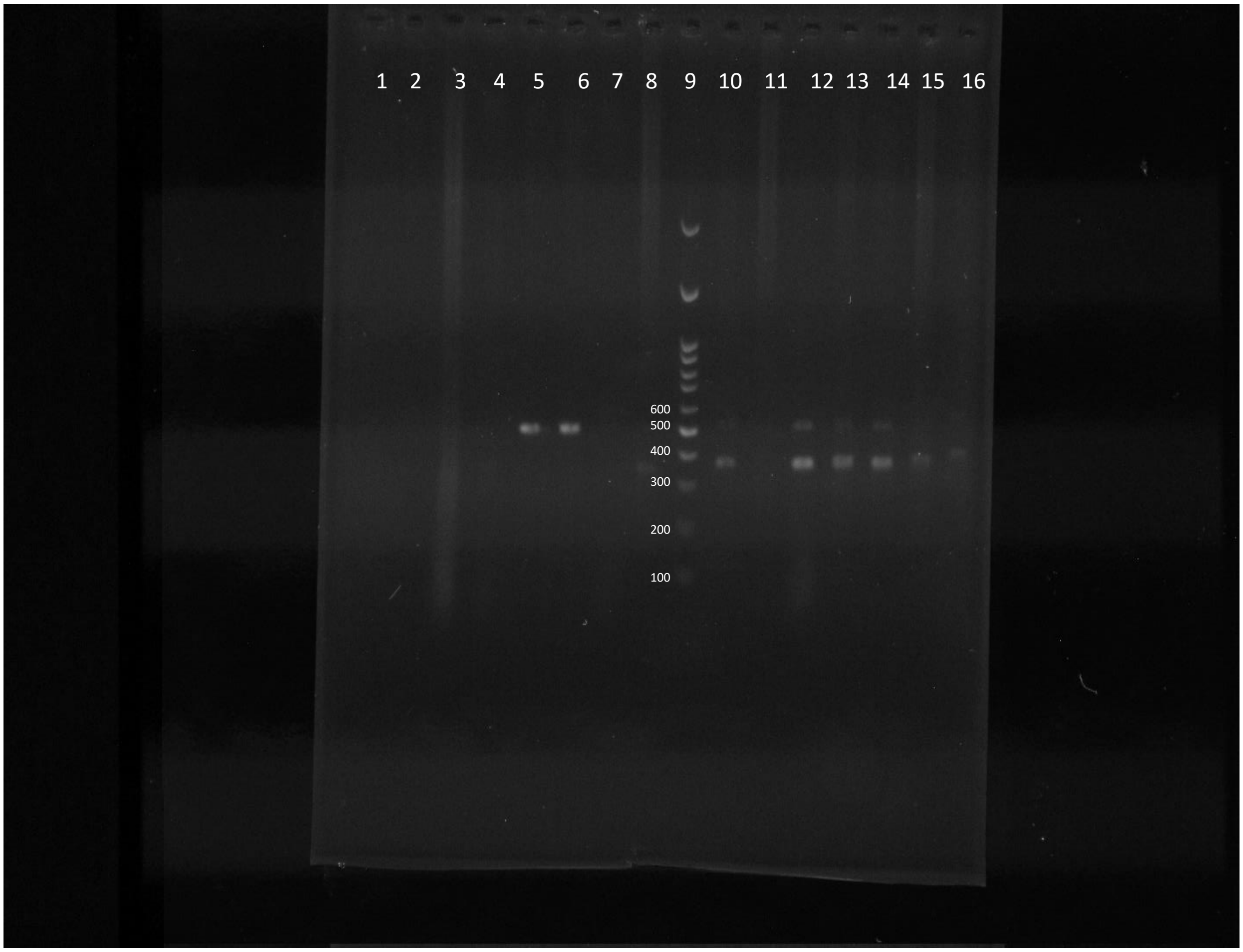


Treatment 4 (Fennel extract)- DNA Ladder (9). 1 and 2 empty, Male embryo: molecular weight of 500 bp (4,5), and female embryo: molecular weight of 500 and 350 bp (8,10,12,13,14,15,16).

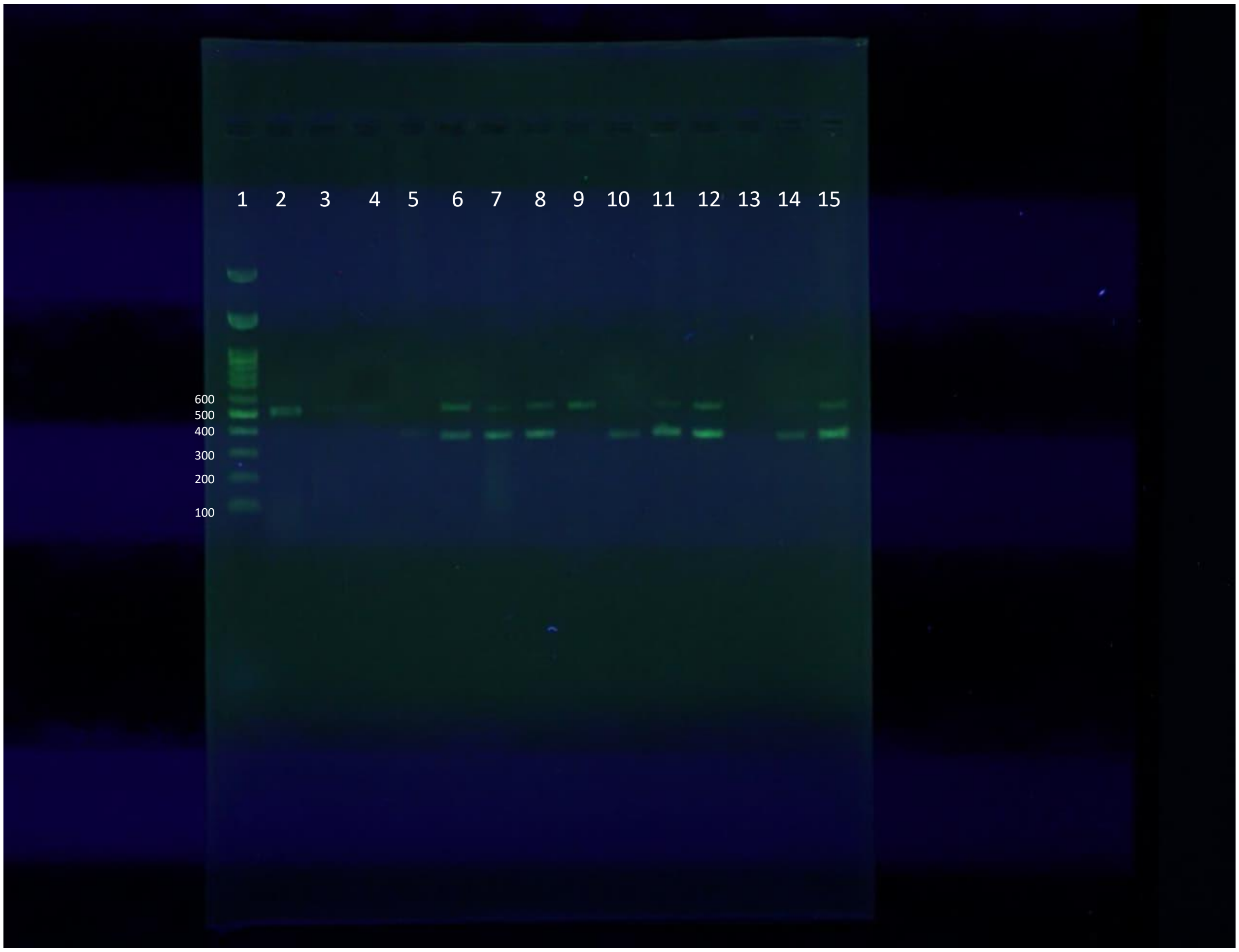


Treatment 5 (Garlic extract)- DNA Ladder (1). Male embryo: molecular weight of 500 bp (2,3,4,9), and female embryo: molecular weight of 500 and 350 bp (5,6,7,8,10,11,12,14,15).

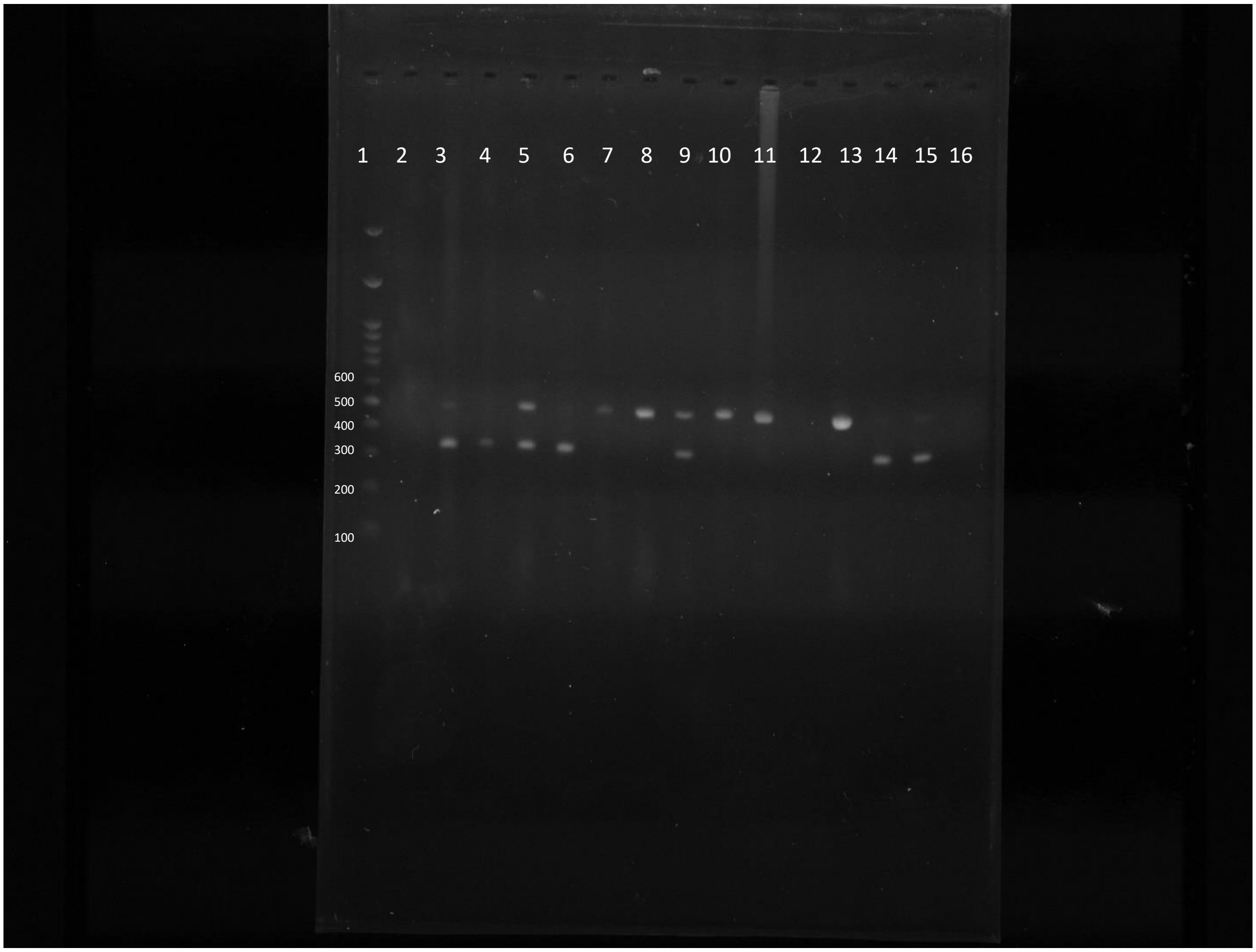


1: DNA Ladder. Numbers 2, 3 and 4 are treatment2 (testosterone), 2 and 3 are the same sample with different concentration of DNA. The band was identified in sample 3

The samples 5, 6, 7 and 8 are treatment 3 (progesterone). 5and 6 are the same sample and 7 and 8 are the same sample. The samples 9 and 10 are treatment 4 (Fennel extract). The samples 11, 13, 14 and 15 are treatment 5 (Garlic extract), The samples 11 and 12 are the same with different DNA concentrations. Number 16: empty

Samples where the band was not clear, PCR was performed with different concentration of DNA and observed again on the gel.
